# Semilobar Holoprosencephaly: Capacious Anomaly in the Cephalad

**DOI:** 10.7759/cureus.9181

**Published:** 2020-07-14

**Authors:** Manikandasamy Veluchamy, Mariappan Murugan

**Affiliations:** 1 Neonatology, NMC Specialty Hospital, Dubai, ARE; 2 Radiodiagnosis, Velammal Medical College Hospital and Research Institute, Madurai, IND

**Keywords:** holoprosencephaly, chiari ii malformation, semilobar, lobar, alobar, prosencephalon, meningomyelocele, arnold chiari

## Abstract

The holoprosencephalies (HPEs) are a group of disorders that are characterized by a failure of differentiation and midline cleavage of the prosencephalon, which usually occurs between days 18 and 28 of gestation. HPE has been divided into three subcategories based on the structural malformation: alobar, semilobar, and lobar HPE. Middle interhemispheric variant (MIH) or syntelencephaly is also considered as a milder variant of HPE. It is estimated to occur in 1/16,000 live births and 1/250 conceptuses. HPE is caused by genetic factors or environmental factors and teratogens. Clinical presentation depends on the severity of the malformation. Severe cases are usually associated with facial abnormalities like hypertelorism or midline facial clefts. HPE is diagnosed prenatally by ultrasound and MRI. Treatment of HPE is supportive and symptomatic. The clinical outcome depends on the severity of HPE and associated medical and neurological complications.

## Introduction

The holoprosencephalies (HPEs) are a group of disorders that are characterized by a failure of differentiation and midline cleavage of the prosencephalon into right and left cerebral hemispheres, which usually occurs between days 18 and 28 of gestation. It is estimated to occur in 1/16,000 live births and 1/250 conceptuses [1].

Holoprosencephaly has been divided into three subcategories based on the structural malformation: alobar, semilobar, and lobar HPE. Middle interhemispheric variant (MIH) or syntelencephaly is also considered as a milder variant of HPE [2]. The forebrain malformations are generally associated with facial anomalies, ranging from anophthalmia, cyclopia, or proboscis in the most severe cases, to midline cleft lip, a simple hypotelorism or even no anomalies in the less severe HPE forms.

Holoprosencephaly is caused by genetic factors or environmental factors and teratogens. HPE is a genetically heterogeneous anomaly and this phenotype is known to be a part of different syndromes or chromosomal anomalies. It can be seen in congenital anomaly syndromes like Smith-Lemli-Opitz and Pallister-Hall syndromes. It has also been noted in association with Patau syndrome (trisomy 13), Edwards syndrome (trisomy 18, less common), and triploidy. Mutations of at least 10 different genetic loci have been implicated in the development of familial HPE: HPE1 on chromosome 21q22.3, HPE2 (SIX3) on 2p21, HPE3 (SHH) on 7q36, HPE4 (TGIF) on 18p, HPE5 (ZIC2) on 13q32, HPE6 at 2q37, HPE7 (PTCH1) at 9q22.3, HPE8 at 14q13, HPE9 (GLI12) at 2q14. All of these genes seem to be involved in the dorsal or ventral induction of the prosencephalon.

Children with HPE have many medical problems like developmental delay, feeding difficulties, epilepsy, temperature instability, heart rate variability, and respiratory abnormalities. Endocrine disorders like diabetes insipidus, adrenal hypoplasia, hypogonadism, thyroid hypoplasia, and growth hormone deficiency are frequent [3].

Anatomy scan done between 18 and 20 weeks of gestation can diagnose HPE antenatally; further fetal MRI can also be done to assess the severity of anomaly. The precise diagnosis regarding the severity of malformations is necessary for genetic counseling and also for termination of pregnancy. Milder HPE cases or microforms may not be recognizable prenatally due to macroscopic normal brain. The prognosis and clinical outcome depend on the severity of HPE and associated medical and neurological complications.

We present two case reports of semilobar HPE. Our first case is a live-born infant with thoracolumbar myelomeningocele and Chiari II malformation associated with semilobar HPE. To the best of our knowledge and available literature evidence, this was the third occurrence in a viable infant. The second case is a live-born infant with a semilobar HPE without any other associated anomalies.

## Case presentation

Case 1

**Figure 1:**
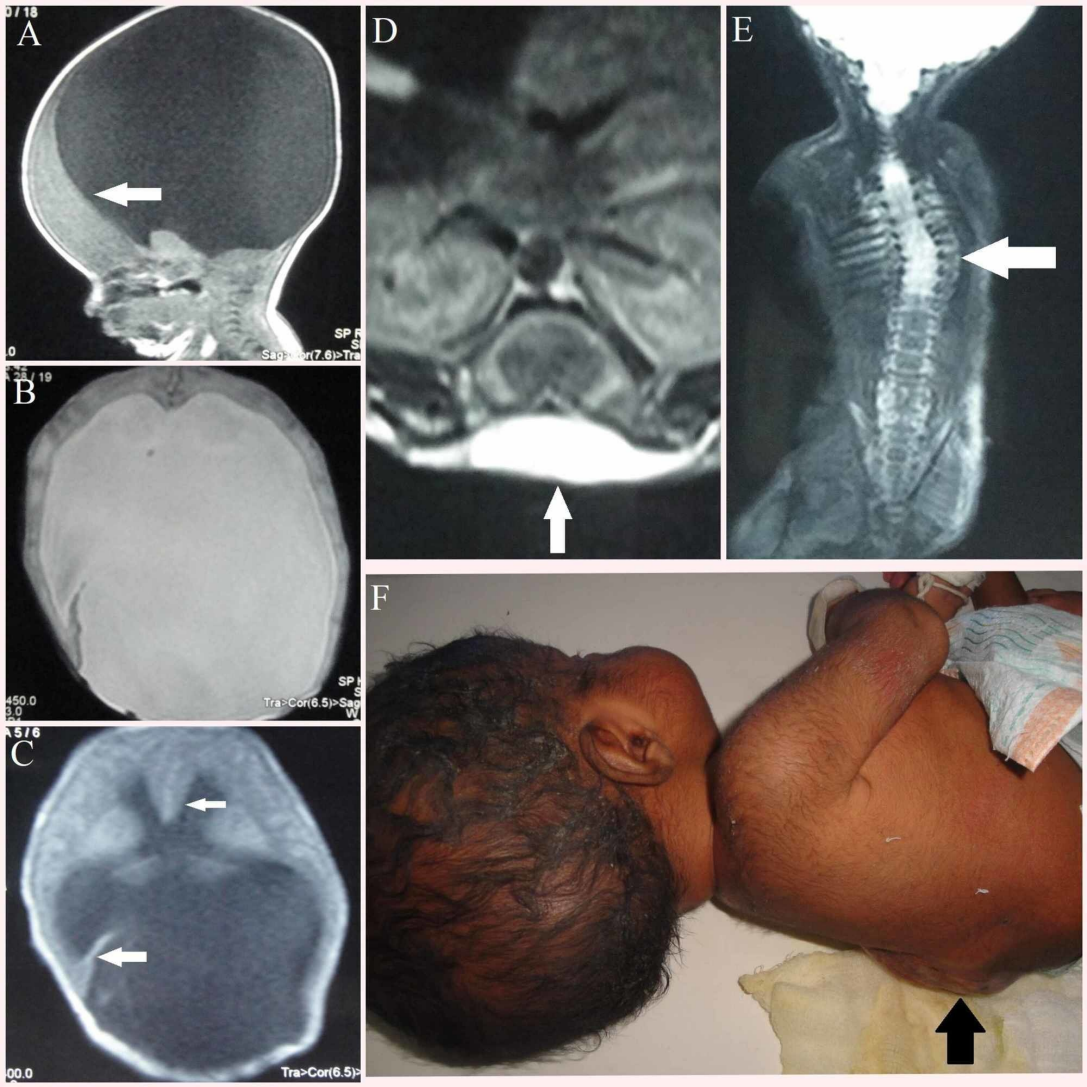
Case 1 – semilobar HPE with Chiari II malformation and meningomyelocele. A) Sagittal T1 weighted image showing (white arrow) severe thinning of frontal lobe of cerebrum with no parieto occipital lobe formation. B) Axial T2 weighted image showing most of the brain parenchyma was replaced by cerebrospinal fluid (CSF). C) Axial T1 weighted image (small horizontal arrow) showing rudimentary anterior falx cerebri with absence of septum pellucidum and agenesis of corpus callosum, (large horizontal arrow) showing incompletely formed tentorium cerebelli. D) Axial T2 weighted image of spine (white arrow) showing open spinal dysraphism. E) Coronal T2 weighted image of spine (white arrow) showing completely dysraphic posterior elements from D6 to L2 vertebra, through which neural elements protruding as sac with hyperintense signals. F) Baby with (black arrow) myelomeningocele in thoracolumbar vertebral region. HPE, holoprosencephaly

A term female baby weighing 2.96 kg was born to a 23-year-old primigravida mother at 38 weeks gestation by normal vaginal delivery. There was no significant antenatal illness and the parents were nonconsanguineous. Antenatal ultrasound was not done. The baby had an Apgar of 8/10 at 1' and 9/ 10 at 5'. She was born with a ruptured meningomyelocele at the lower thoracic vertebral level, without any dysmorphic features, a length of 48 cm, and a head circumference of 37 cm. The baby was hemodynamically stable without any movement of both lower limbs. Cardiovascular and respiratory systems were normal. MRI brain was done which showed rudimentary anterior falx cerebri with incompletely formed interhemispheric fissure, absence of septum pellucidum with agenesis of the corpus callosum, severe thinning of cerebrum without parieto-occipital lobe formation, dysplastic unfused thalamus, platybasia with horizontally placed clivus, posterior fossa appears smaller with dysplastic cerebellum and brainstem, and there is herniation of cerebellum and brainstem with widened foramen magnum (Figure 1). MRI spine also was done which revealed thin and dysplastic spinal cord with tethering of cord noted at sixth thoracic vertebral level, spinal dysraphism with defective posterior elements at seventh cervical vertebral level through which neural elements are protruding into the sac, and this sac is seen continuous with bony spinal canal deformity which is hypointense in T1 weighted image and hyperintense in T2 weighted image and it extends from D6 to L2 level (Figure 1).

The MRI findings were consistent with a case of semilobar HPE with Arnold Chiari type II malformation. The chromosomal analysis report was within normal limits. Parental counseling was done regarding the baby’s poor prognosis. The baby was released from the hospital as the parents did not want to continue further management.

Case 2

**Figure 2:**
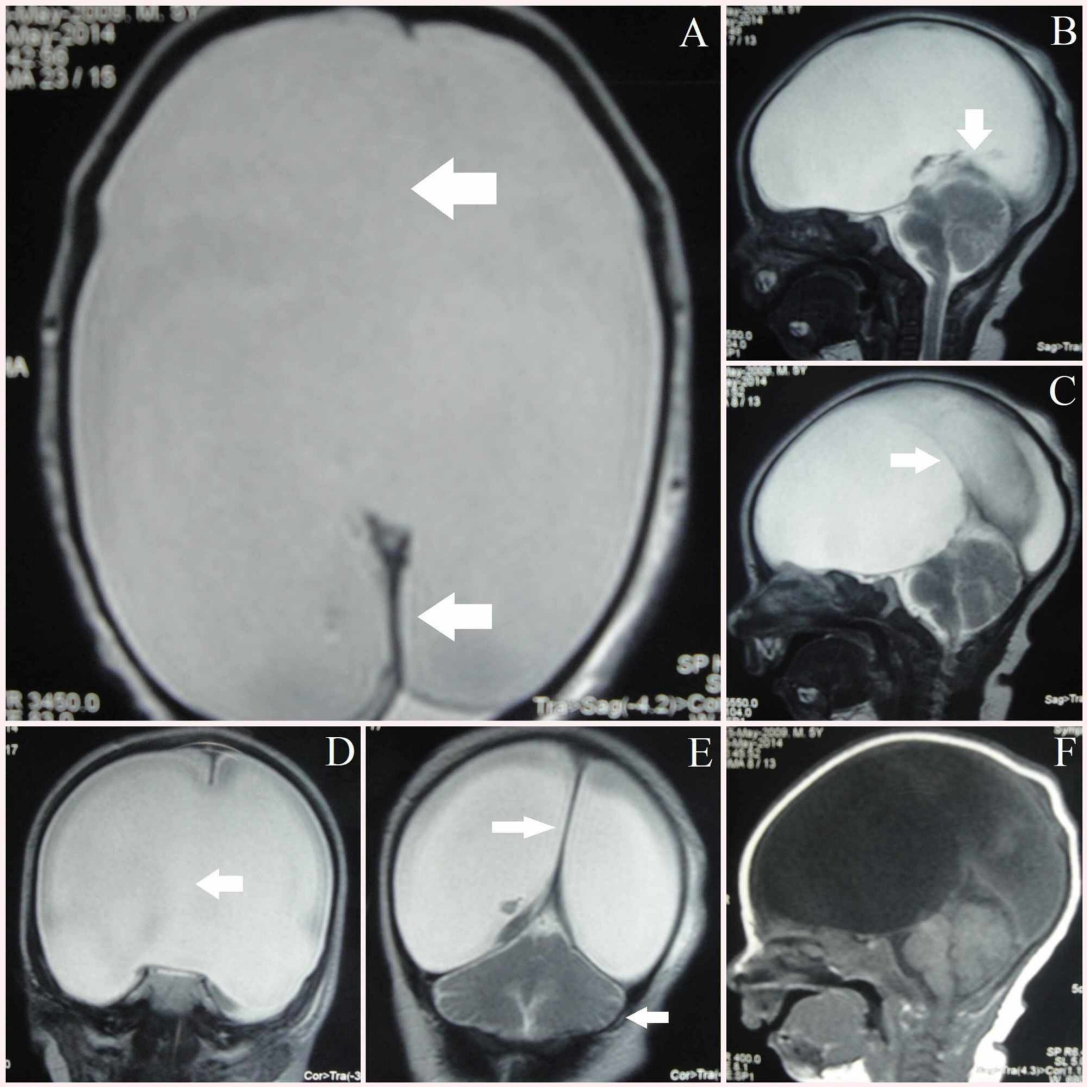
Case 2 – semilobar prosencephaly. A) Axial T2 weighted image showing (big leftward arrow) absent falx cerebri, absent midline structures, monoventricle and (small leftward arrow) rudimentary posterior interhemispheric fissure B) Sagittal T2 weighted image showing (downward arrow) normally appearing cerebellum and posterior fossa structures C) Sagittal T2 weighted image showing (rightward arrow) rudimentary posterior interhemispheric fissure, absence of midline structures anteriorly D) Coronal T2 weighted image showing monoventricle with (horizontal leftward arrow) absent midline structures E) Coronal T2 weighted image showing (large rightward arrow) rudimentary posterior interhemispheric fissure dividing cerebral hemispheres with (small leftward arrow) normal cerebellum F) Sagittal T1 weighted image corresponding to image C

A term male baby weighing 3.9 kg was born to a 38-year-old mother at 40 weeks gestation by normal vaginal delivery. He was born to a third-degree consanguineous parent without any significant antenatal illness. Antenatal ultrasound was not done. The baby had an Apgar of 8/10 at 1' and 9/10 at 5'. The baby developed poor feeding and lethargy on day 2 of life, evaluated for neonatal sepsis, and initial investigations for sepsis were within normal limits. MRI was done which showed monoventricle with rudimentary posterior interhemispheric fissure and incomplete falx cerebri. There is an absence of corpus callosum, cavum septum pellucidum, and anterior fossa midline structures, posterior fossa and both cerebellar hemispheres appear normal (Figure 2).

The MRI features suggest that this is a semilobar type of HPE. The baby showed gradual improvement and was released from the hospital on day 7 of life.

## Discussion

The arrest of the division of the prosencephalon is thought to occur during the period of brain division between the third and fifth weeks of gestation. HPE is an uncommon anomaly with an incidence of 1 in 16,000 live births.

The most severe form of HPE, alobar HPE is characterized by a complete lack of separation of the prosencephalon, with a monoventricle, fused thalami, absent corpus callosum, and associated midline facial anomalies such as hypotelorism, cyclopia, micro and enopthalmia, and cleft lip/palate. In general, although the degree of facial dysmorphism is said to parallel the degree of intracranial malformation, the opposite is not always true. These babies have a poor prognosis and they may not survive beyond infancy.

The least severe form of HPE, lobar HPE in which greater separation of cerebral hemispheres and lateral ventricles present. The variable fusion of the thalami and incomplete formation of the corpus callosum is also noted. In contrast to the alobar type, facial deformities are usually not present. These children may survive to adulthood but are mentally retarded.

The intermediate arrest of division results in semilobar HPE, with the partial formation of the lateral ventricles, fused thalami, and absent corpus callosum. These babies may or may not have facial anomalies and survival lies between that associated with the alobar and lobar types [4].

In the early 1890s, Professor Hans Chiari, an Austrian anatomopathologist at the German University in Prague, Czechoslovakia, described the congenital anomalies that would later be named Chiari malformation types I-IV. Chiari malformations are a type of rhombencephalic anomalies. These anomalies are characterized by downward elongation or displacement of the cerebellar tonsils or the vermis into the cervical spinal canal [5].

Chiari malformation type I (CM I) is the least severe type, which involves caudal displacement of cerebellar tonsil inferior to the plane of foramen magnum by 3-5 mm. This CM I is associated with various anatomical findings involving the skull, spine, meninges, and spinal cord. Hydrocephalus is infrequent in this type, seen only in 10% of children with this malformation. Syringomyelia is seen in 45%-75% of patients with CM I. CM I is mostly congenital and presents in childhood or early adulthood. The presentation is diverse. Headache is the most common presenting feature. It is classically described as a sub-occipital headache or neck pain triggered or exacerbated by Valsalva-like maneuvers (such as coughing or straining) [6-7].

Chiari malformation type II (CM II) is the most frequent form. It is also known as ‘classic’ or ‘Arnold-Chiari’ malformation. This is a pancerebral malformation, and the most striking feature of this malformation involves the posterior fossa. CM II is a broad group of malformation involving both neuroectoderm and mesoderm. Neuroectodermal abnormalities include caudal displacement of the cerebellar tonsils and vermis, as well as the caudal brainstem (medulla and, variably, the pons), through an enlarged foramen magnum into the cervical spinal canal. Mesodermal abnormalities involve the membranous skull and basicranium which includes a small posterior fossa, a low-lying tentorium with a much-enlarged tentorial incisura, a foreshortened clivus, and scalloping of the petrous bone.

This Chiari II malformation invariably associated with meningomyelocele. CM II usually presents at birth and is now increasingly identified in routine anatomy scan prenatally. Infants may have neurogenic dysphagia (presenting as poor feeding, choking, regurgitation, aspiration pneumonia), vocal cord paralysis (causing altered phonation, a hoarse or high-pitched cry), impaired respiratory drive (presenting as apnoea or respiratory arrest), or stridor. Hydrocephalus is more frequent in this type, which will produce more characteristic signs of increased intracranial pressure [7-8].

Chiari malformation type III (CM III) is a rare form. Chiari originally described it as a cervical cerebellar hydrocephalocele, which entails herniation of part of the cerebellum in an occipital, cervical, or occipital-cervical meningocele along with caudal displacement of the medulla. Hydrocephalus is present in half of the cases and is of the obstructive type.

Chiari malformation type IV (CM IV) is the least frequent but most severe form of CM, characterized by an incomplete or undeveloped cerebellum (cerebellar hypoplasia or aplasia) and alterations of the pons with a ‘pigeon breast’ deformity of the brainstem [5].

Chiari II malformation with co-existing myelomeningocele comprises one of the most common severe central nervous system (CNS) malformations. HPE is a less common but equally complex CNS malformation. As per the available literature, Chiari II malformation and HPE differ with respect to the timing of the teratogenic insult and the proposed developmental aberration. HPE occurs because of early embryonic insult, suggesting that the malformations were incompatible with later fetal development. The developmental aberrations that result in Chiari II malformation occur later in embryological development or in early fetal life. The coexistence of these malformations HPE and Chiari II is usually incompatible with life and presumably accounts for the rarity with which these malformations are reported to occur together [9].

Britton reported a case of semilobar HPE with associated Arnold Chiari variant, in which a preterm female baby was born to a mother with pre-eclampsia. The baby was found to have macrocephaly with thoracolumbar meningomyelocele and distorted left hemithorax. Neuroimaging revealed partial anterior separation of a dominant monoventricle into two lateral ventricles, fused thalami, partial falx, and thin cerebral cortex. Baby had died after 24 hours of life in view of discontinuation of supportive measures [4].

Rollins et al. reported a similar case in the live-born infant which later presented at 13 months of age with microcephaly, seizures, and global development delay [9]. Mittelbronn et al. reported a similar case in their post-mortem neuropathological findings [10].

Other cases of HPE with associated anomalies were reported by Harlow et al. in which HPE was associated with spinal anomaly caudal regression in a live-born term male infant and Chen et al. reported a stillborn female infant with a lumbar meningocele, cebocephaly, and alobar HPE [11-12].

Holoprosencephaly and Chiari II malformation share some common morphological abnormalities which include dysplasias of cytoarchitecture, dysgenetic or absent corpus callosum and midline structures, and hydrocephalus of variable severity. These are nonspecific abnormalities and occur in association with numerous syndromic and nonsyndromic cerebral malformations [13].

Many theories have been proposed to explain the causes of Chiari malformation, but none of the cause is clear. McLone and Knepper combined these theories into a ‘unified theory’ of Chiari malformation. In this concept, they proposed that incomplete closure of the neural tube and the resultant mechanical derangements are the main cause for the intracranial abnormalities found in patients with myelomeningoceles and Chiari II malformations. The presence of both an open neural tube defect and incomplete spinal occlusion causes loss of cerebrospinal fluid (CSF) troughs resulting in a subsequent drop in intracranial pressure. CSF drains through the central canal and is therefore not retained in the ventricular system. The absence of the ventricular CSF driving force during fetal development results in poor cranial vault expansion, resulting in a small posterior fossa. The unexpectedly narrowed posterior fossa leads to caudal displacement of the brainstem and cerebellum through the foramen magnum. This theory also explains the development of hydrocephalus due to overcrowding of the posterior; with CSF outflow blocked or impaired at the foramina of Luschka and Magendie, progressive ventriculomegaly ensues [14].

There are different opinions regarding the cause of HPE. According to Probst, severe median prosencephalic dysgenesis under the organizing inﬂuence of prechordal mesoderm results in HPE, whereas Muller and O’Rahilly concluded that an early defect in the mesencephalic neural crest may be the genesis of HPE. Barkovich and Quint suggested that lack of formation of the interhemispheric ﬁssure is the pivotal anomaly in HPE and that it results from the paucity of mesenchyme that normally invests the deepening interhemispheric groove separating the developing cerebral hemispheres [2].

 The anomalies in Chiari II malformation are characterized by mesodermal dysplasia and premature condensation of the basilar skull, small and dysplastic lower cranial nerve ganglia, deﬁcient tentorium cerebelli, hypoplastic and dysmorphic cerebellum, and thickened basal meninges. These anomalies all are attributable to defective or deﬁcient mesenchyme. The role of mesenchyme in the pathogenesis of Chiari II malformation is meagrely explained in the literature.

The importance of the supportive role of mesenchyme in the initial phase of neurulation has been stressed by embryologists who note that mesenchymal deﬁciency may cause neural tube defects. During early embryogenesis, mesenchyme cells begin to surround the hindbrain at the time of neural tube closure and continue to spread to the midbrain and the forebrain levels. The meninges, tentorium, and cranial nerve ganglia are all derived from mesenchyme [15].

## Conclusions

Anatomy scan or Level II ultrasound is done at 20 weeks of gestation to evaluate fetal anatomy and is an important part of prenatal care. The anatomy scan is used for early detection of anomalies like HPE and Chiari II malformation, which can guide in parental counseling and also in the prevention of babies born with severe anomalies, thereby decreasing the morbidity and mortality associated with these anomalies.

Even though HPE and Chiari II malformation differs in their incidence rate, morphology, and timing of insult, they may co-exist. Mesenchymal abnormality can lead to HPE, similarly defective or deficient mesenchyme may cause Chiari II malformation. The prognosis and outcome of HPE depend on the severity of HPE and the presence of associated medical and neurological complications.

## References

[1] Dubourg C, Bendavid C, Pasquier L, Henry C, Odent S, David V (2007). Holoprosencephaly. Orphanet J Rare Dis.

[2] Barkovich AJ, Quint DJ (1993). Middle interhemispheric fusion: an unusual variant of holoprosencephaly. Am J Neuroradiol.

[3] Barkovich AJ, Raybaud CA (2012). Congenital malformations of the brain and skull. Pediatric Neuroimaging (5th Edition).

[4] Britton CA (1989). Semilobar holoprosencephaly with associated Arnold-Chiari variant. J Clin Ultrasound.

[5] Mancarella C, Delfini R, Landi A (2019). Chiari malformations. Acta Neurochir Suppl.

[6] Azahraa Haddad F, Qaisi I, Joudeh N (2018). The newer classifications of the chiari malformations with clarifications: an anatomical review. Clin Anat.

[7] Piper RJ, Pike M, Harrington R, Magdum SA (2019). Chiari malformations: principles of diagnosis and management. BMJ.

[8] McLone DG, Dias MS (2003). The Chiari II malformation: cause and impact. Childs Nerv Syst.

[9] Rollins N, Joglar J, Perlman J (1999). Coexistent holoprosencephaly and Chiari II malformation. Am J Neuroradiol.

[10] Mittelbronn M, Beschorner R, Capper D, Haist M, Meyermann R, Meyer-Wittkopf M (2006). Coincidence of semilobar holoprosencephaly and Chiari II malformation: correlation of prenatal diagnostics and neuropathologic findings. J Child Neurol.

[11] Harlow CL, Partington MD, Thieme GA (1995). Lumbosacral agenesis: clinical characteristics, imaging, and embryogenesis. Pediatr Neurosurg.

[12] Chen CP, Shih SL, Liu FF, Jan SW (1997). Cebocephaly, alobar holoprosencephaly, spina bifida, and sirenomelia in a stillbirth. J Med Genet.

[13] Gilbert JN, Jones KL, Rorke LB, Chernoff GF, James HE (1986). Central nervous system anomalies associated with meningomyelocele, hydrocephalus, and the Arnold-Chiari malformation: reappraisal of theories regarding the pathogenesis of posterior neural tube closure defects. Neurosurgery.

[14] McLone DG, Knepper PA (1989). The cause of Chiari II malformation: a unified theory. Pediatr Neurosci.

[15] Dasgupta K, Jeong J (2019). Developmental biology of the meninges. Genesis.

